# Supplementary material for: Spatial Variation in Stream-Water Bacterial Communities Across Sites Differing in Wild Chinese Giant Salamander Detection Frequency
Source: Biology (Basel). 2026 Sep 18;15(18):1647. doi: 10.3390/biology15181647 (PMC13604818; doi:10.3390/biology15181647)
Supplement: Supplementary file 1 [file biology-15-01647-s001.zip › Data Related to “Spatial Variation in Stream-Water Bacterial Communities across Sites Differing in Wild Chinese Giant Salamander Detection Frequency”/Microbial Data/report/src/hyperlinks/Advanced_ReadMe_EN.html]

| result |  |
| --- | --- |
| \|-- 07.CorrelationAnalysis | 【Correlation Analysis】 |
| \| \|-- network | 【 network results】 |
| \| \| \|-- group1/... | 【network results for group】 |
| \| \| \| \|-- \*/dot/genus.{png,svg} | 【graphical display of network results】 |
| \| \| \| \|-- \*/dot/genus.spearman.index.list | 【the edge of network】 |
| \| \| \| \|-- \*/dot/node.xls | 【the node of network】 |
| \| \| \| \|-- \*/dot/igraph.calculate.txt | 【Display of key parameters of the network，such as Network diameter(ND),Average degree(AD),etc】 |
| \| \| \|-- sample/... | 【network results for samples】 |
| \| \| \| \|-- dot/genus.{png,svg} | 【graphical display of network results】 |
| \| \| \| \|-- dot/genus.spearman.index.list | 【the edge of network】 |
| \| \| \| \|-- dot/node.xls | 【the node of network】 |
| \| \| \| \|-- dot/igraph.calculate.txt | 【Display of key parameters of the network，such as Network diameter(ND),Average degree(AD),etc】 |
| \| \|-- network3D | 【 network3D results】 |
| \| \| \|-- group1/... | 【network3D results for group】 |
| \| \| \| \|-- \*/cytoplot.csv | 【the data for Cytoscape】 |
| \| \| \| \|-- \*/edge.xls | 【the edge of network3D】 |
| \| \| \| \|-- \*/node.xls | 【the node of network3D】 |
| \| \| \| \|-- netfig | 【 files of network3D Web page】 |
| \| \| \|-- sample/... | 【network3D results for samples】 |
| \| \| \| \|-- cytoplot.csv | 【the data for Cytoscape】 |
| \| \| \| \|-- edge.xls | 【the edge of network3D】 |
| \| \| \| \|-- node.xls | 【the node of network3D】 |
| \| \| \| \|-- netfig | 【 files of network3D Web page】 |
| \|-- 08.FunctionPrediction | 【Function Prediction results】 |
| \| \|-- picrust2 | 【 picrust2 prediction results】 |
| \| \| \|-- predicted | 【 picrust2（COG,EC,KO,PFAM,TIGRFAM,pathway）prediction results】 |
| \| \| \| \|-- \*\_metagenome\_descrip.tsv | 【（COG,EC,KO,PFAM,TIGRFAM,pathway）prediction results】 |
| \| \| \| \|-- \*\_seqtab\_norm.tsv.gz | 【Feature Table Based on 16S Copy Number Correction(excluding pathways\_out)】 |
| \| \| \| \|-- \*\_weighted\_nsti.tsv.gz | 【NSTI weight for each sample(excluding pathways\_out)】 |
| \| \| \|-- tables | 【relative (absolute) abundance of predicted results】 |
| \| \| \| \|-- sample/group1/... | 【relative (absolute) abundance of sample and group prediction results】 |
| \| \| \| \| \|-- picrust2.{sample,group}.\*.absolute.xls | 【absolute abundance of predicted results for each database of sample and group)】 |
| \| \| \| \| \|-- picrust2.{sample,group}.\*.relative.xls | 【relative abundance of predicted results for each database of sample and group】 |
| \| \| \|-- barplot | 【Barplot of function annotation】 |
| \| \| \|-- heatmap | 【Heatmap of function annotation】 |
| \| \| \|-- venn | 【Venn diagram of function annotation】 |
| \| \| \|-- flower | 【Flower diagram of function annotation】 |
| \| \| \|-- pca | 【PCA analysis of function annotation】 |
| \| \| \|-- ttest | 【T-test analysis of function annotation】 |
| \| \|-- picrust | 【 Picrust Function Prediction】 |
| \| \| \|-- predicted | 【 KEGG of Function Prediction】 |
| \| \| \| \|-- KEGG\_metagenome\_predictions.{biom,txt} | 【 KEGG of Function Prediction】 |
| \| \| \| \|-- predicted\_metagenomes.KEGG\_L\*.txt | 【 KEGG（1,2,3）levels function prediction】 |
| \| \| \|-- tables | 【relative (absolute) abundance of predicted results】 |
| \| \| \| \|-- sample/group1/... | 【relative (absolute) abundance of sample and group prediction results】 |
| \| \| \| \| \|-- picrust.{sample,group}.\*.absolute.xls | 【absolute abundance of sample and group prediction results（1,2,3,k）】 |
| \| \| \| \| \|-- picrust.{sample,group}.\*.relative.xls | 【absolute abundance of sample and group prediction results（1,2,3,k）】 |
| \| \| \|-- barplot | 【Barplot of function annotation】 |
| \| \| \|-- heatmap | 【Heatmap of function annotation】 |
| \| \| \|-- venn | 【Venn diagram of function annotation】 |
| \| \| \|-- flower | 【Flower diagram of function annotation】 |
| \| \| \|-- pca | 【PCA analysis of function annotation】 |
| \| \| \|-- ttest | 【T-test analysis of function annotation】 |
| \| \|-- tax4fun | 【 Tax4Fun Function Prediction】 |
| \| \| \|-- predicted | 【 KEGG of Function Prediction】 |
| \| \| \| \|-- KEGG\_metagenome\_predictions.txt | 【 KEGG of Function Prediction】 |
| \| \| \| \|-- predicted\_metagenomes.KEGG\_L\*.txt | 【 KEGG（1,2,3）levels function prediction】 |
| \| \| \|-- tables | 【relative (absolute) abundance of predicted results】 |
| \| \| \| \|-- sample/group1/... | 【relative (absolute) abundance of sample and group prediction results】 |
| \| \| \| \| \|-- tax4fun.{sample,group}.\*.absolute.xls | 【absolute abundance of sample and group prediction results（1,2,3,k）】 |
| \| \| \| \| \|-- tax4fun.{sample,group}.\*.relative.xls | 【relative abundance of sample and group prediction results（1,2,3,k）】 |
| \| \| \|-- barplot | 【Barplot of function annotation】 |
| \| \| \|-- heatmap | 【Heatmap of function annotation】 |
| \| \| \|-- venn | 【Venn diagram of function annotation】 |
| \| \| \|-- flower | 【Flower diagram of function annotation】 |
| \| \| \|-- pca | 【PCA analysis of function annotation】 |
| \| \| \|-- ttest | 【T-test analysis of function annotation】 |
| \| \|-- bugbase | 【 bugbase Function Prediction】 |
| \| \| \| \|-- normalized\_otus | 【normalized OTU tables】 |
| \| \| \| \|-- otu\_contributions | 【Phenotypic prediction results】 |
| \| \| \| \| \|-- \*.{pdf,png} | 【barplot of phenotypic prediction results】 |
| \| \| \| \| \|-- contributing\_otus.txt | 【table of phenotypic prediction results】 |
| \| \| \| \| \|-- otu\_table.relative.xls | 【relative abundance of Phenotypic Prediction Results】 |
| \| \| \| \|-- predicted\_phenotypes | 【boxplot of phenotype prediction results】 |
| \| \| \| \| \|-- \*.{pdf,png} | 【boxplot of phenotype prediction results】 |
| \| \| \| \| \|-- \*\_stats.txt | 【parameters such as mean, median, and standard deviation for boxplot of phenotype prediction results】 |
| \| \| \| \| \|-- predictions.{txt,xls} | 【table of Phenotypic Abundance】 |
| \| \| \| \|-- thresholds | 【table of thresholds】 |
| \| \| \| \| \|-- variances.txt | 【results under different thresholds】 |
| \| \| \| \| \|-- thresholds\_used.txt | 【threshold used for phenotype prediction analysis】 |
| \| \| \| \| \|-- \*.{pdf,png} | 【relative abundance changes of 9 phenotypes under different thresholds】 |
| \| \|-- funguild | 【Funguild】 |
| \| \| \|-- predicted | 【 funguild function prediction】 |
| \| \| \| \|-- funguild.sample.total.absolute.xls | 【 funguild function prediction results】 |
| \| \| \| \|-- funguild.sample.total.absolute.{guild, mode} | 【 funguild（guild,mode）Function Prediction】 |
| \| \| \|-- tables | 【relative (absolute) abundance of predicted results】 |
| \| \| \| \|-- sample/group1/... | 【relative (absolute) abundance of sample and group prediction results】 |
| \| \| \| \| \|-- funguild.{sample,group}.\*.absolute.xls | 【absolute of sample and group prediction results（total,guild,mode)】 |
| \| \| \| \| \|-- funguild.{sample,group}.\*.relative.xls | 【relative abundance of sample and group prediction results（total,guild,mode)】 |
| \| \| \|-- barplot | 【Barplot of function annotation】 |
| \| \| \|-- heatmap | 【Heatmap of function annotation】 |
| \| \| \|-- venn | 【Venn diagram of function annotation】 |
| \| \| \|-- flower | 【Flower diagram of function annotation】 |
| \| \| \|-- pca | 【PCA analysis of function annotation】 |
| \| \| \|-- ttest | 【T-test analysis of function annotation】 |
| \| \|-- faprotax | 【 faprotax function prediction】 |
| \| \| \|-- predicted | 【 faprotax function prediction】 |
| \| \| \| \|-- faprotax.report.txt | 【 faprotax function prediction report】 |
| \| \| \| \|-- faprotax.sample.anno.absolute.xls | 【faprotax function prediction results】 |
| \| \| \|-- tables | 【relative (absolute) abundance of predicted results】 |
| \| \| \| \|-- sample/group1/... | 【relative (absolute) abundance of sample and group prediction results】 |
| \| \| \| \| \|-- faprotax.{sample,group}.anno.absolute.xls | 【absolute abundance of sample and group prediction results】 |
| \| \| \| \| \|-- faprotax.{sample,group}.anno.relative.xls | 【relative abundance of sample and group prediction results】 |
| \| \| \|-- barplot | 【Barplot of function annotation】 |
| \| \| \|-- heatmap | 【Heatmap of function annotation】 |
| \| \| \|-- venn | 【Venn diagram of function annotation】 |
| \| \| \|-- flower | 【Flower diagram of function annotation】 |
| \| \| \|-- pca | 【PCA analysis of function annotation】 |
| \| \| \|-- ttest | 【T-test analysis of function annotation】 |
| \|-- 09.EnvironmentFactor | 【Environmental association analysis】 |
| \| \|--CCA | 【Canonical Correspondence Analysis (CCA) and Redundancy Analysis (RDA)】 |
| \| \| \|--CCA\_standard | 【CCA and RDA including all environmental factors】 |
| \| \| \|--CCA\_FitENV | 【CCA and RDA of environmental factors screened by envfit】 |
| \| \| \|--CCA\_VIF | 【CCA and RDA of environmental factors screened by VIF】 |
| \| \| \|--CCA\_BioENV | 【CCA and RDA of environmental factors screened by BioENV】 |
| \| \| \|--BioENV.txt | 【Environmental factor combinations obtained through Bioenv screening】 |
| \| \| \|--VIF.txt | 【Environmental factor combinations obtained through VIF screening】 |
| \| \| \|--selected\_FitENV.list | 【Environmental factor combinations obtained through FitENV screening】 |
| \| \|--mantelTest | 【mantel\_test results at each taxonomic level (total,k,p,c,o,f,g,s)】 |
| \| \|--spearman | 【spearman results at each taxonomic level (total,k,p,c,o,f,g,s)】 |
| \| \|--vpa | 【Variance Partitioning Canonical Correspondence Analysis (VPA)】 |
| \| \| \|--VPA1.{png,svg} | 【VPA results】 |
|  |  |

`Tips:`

- This section is the Readme for the advanced analysis.

  ​

---

```
```
xxxxxxxxxx
```


```
                           If you have any questions, please consult the project sales or operation.
```
```
